# Next-generation-based targeted sequencing as an efficient tool for the study of the genetic background in Hirschsprung patients

**DOI:** 10.1186/s12881-015-0235-5

**Published:** 2015-10-05

**Authors:** Berta Luzón-Toro, Laura Espino-Paisán, Raquel Ma. Fernández, Marta Martín-Sánchez, Guillermo Antiñolo, Salud Borrego

**Affiliations:** Department of Genetics, Reproduction and Fetal Medicine, Institute of Biomedicine of Seville (IBIS), University Hospital Virgen del Rocío/CSIC/University of Seville, Seville, Spain; Centre for Biomedical Network Research on Rare Diseases (CIBERER), Seville, Spain

**Keywords:** Genetics, Hirschsprung disease, NGS panel, Phenotype

## Abstract

**Background:**

The development of next-generation sequencing (NGS) technologies has a great impact in the human variation detection given their high-throughput. These techniques are particularly helpful for the evaluation of the genetic background in disorders of complex genetic etiology such as Hirschsprung disease (HSCR). The purpose of this study was the design of a panel of HSCR associated genes as a rapid and efficient tool to perform genetic screening in a series of patients.

**Methods:**

We have performed NGS-based targeted sequencing (454-GS Junior) using a panel containing 26 associated or candidate genes for HSCR in a group of 11 selected HSCR patients.

**Results:**

The average percentage of covered bases was of 97 %, the 91.4 % of the targeted bases were covered with depth above 20X and the mean coverage was 422X. In addition, we have found a total of 13 new coding variants and 11 new variants within regulatory regions among our patients. These outcomes allowed us to re-evaluate the genetic component associated to HSCR in these patients.

**Conclusions:**

Our validated NGS panel constitutes an optimum method for the identification of new variants in our patients. This approach could be used for a fast, reliable and more thorough genetic screening in future series of patients.

**Electronic supplementary material:**

The online version of this article (doi:10.1186/s12881-015-0235-5) contains supplementary material, which is available to authorized users.

## Background

Hirschsprung disease (HSCR, OMIM 142623) is a developmental disorder occurring in 1 of 5.000 live births. It is characterized by the absence of ganglion cells along variable lengths of the distal gastrointestinal tract, which results in tonic contraction of the aganglionic colon segment and functional intestinal obstruction. Such aganglionosis is associated with a delay in the entry of neural crest-derived cells into the foregut, as well as a deferred progression of enteric neural crest cells along the gut [1–5]. Based on the length of the aganglionic region, patients could be classified as short-segment (S-HSCR: aganglionosis up to the upper sigmoid colon, 80 % of cases), long-segment (L-HSCR: aganglionosis up to the splenic flexure and beyond, 17 % of cases) and total colonic aganglionosis forms (TCA, 3 % of cases) [1]. HSCR most commonly presents sporadically with reduced penetrance and male predominance, although it can be also familial with an autosomal dominant or autosomal recessive model of inheritance. HSCR occurs as an isolated trait in 70 % of cases and it is associated with other congenital malformation syndromes in the remaining 30 % [1, 3, 4].

Therefore, HSCR is regarded as a disorder with complex genetic basis, in which the contribution of several different loci acting in an additive or multiplicative manner is usually required to cause the disease. The *RET* proto-oncogene is the major susceptibility gene for HSCR since more than 80 % of identified mutations associated with HSCR are located in this gene, including both coding and noncoding variants [6–8]. Mutations in *RET* coding sequence account for up to 50 % or 7–20 % of familial and sporadic cases, respectively [1]. Other genes encoding members of a variety of signalling pathways related to enteric nervous system (ENS) development, have been also reported to be related to HSCR (*GDNF, NRTN, PSPN, EDNRB, EDN3, ECE1, NTF3, NTRK3, SOX10, PHOX2B, L1CAM, ZFHX1B, KIAA1279, TCF4, PROK1, PROKR1, PROKR2, GFRA1, NRG1, SEMAPHORIN 3A, SEMAPHORIN 3C* and *SEMAPHORIN 3D*). However, mutations in these genes only explain the minority forms of L-HSCR/TCA or syndromic forms of the disease [9–15].

The development of next-generation sequencing (NGS) technologies has a great impact in human mutation detection procedures given their high throughput nature. In the last 10 years we have witnessed a tremendous increase in sequencing speed paralleled by costs falling dramatically by 10.000–100.000 fold compared to the classical Sanger method [16–19].

The 454-GS Junior (Roche) is a NGS sequencer that leads to a rapid sample processing. In 2012, a study of three class III semaphorin as candidate genes based on amplicon sequencing (454-GS Junior Platform) was performed in 47 HSCR samples. They reported 37 sequence variants, where 10 were unique to HSCR patients, including 5 missense mutations in these three genes that may be potentially involved in the pathogenesis of HSCR [11]. More recently, PCR-based RainDance technology and 454 FLX sequencing have been applied to analyze 62 genes in 20 Chinese HSCR patients and 20 Chinese non HSCR controls, reporting 5 rare damaging variants likely involved in the disease [20].

Here, we have used the 454 GS-Junior Platform to perform NGS-based targeted sequencing to validate the design of our panel. With such purpose, we selected a group of 11 patients carrying a total of 18 different variants, previously identified by Sanger method, in any of the genes included in the panel. After panel validation, we determined the set of candidate variants carried by our patients in these HSCR-associated genes.

## Methods

### Patients and control subjects

Our study involved a total of 11 Spanish HSCR index patients, comprising a male: female ratio equal to 10:1 with different phenotypic features (two with TCA, four with L-HSCR, four with S-HSCR and one with not available data) (Table 1). All patients were referred to our Department of Genetics, Reproduction and Fetal Medicine. Additionally, we had a total of 26 DNA samples from available family members of our patients that were used to perform subsequent segregation analysis of the new identified variants.

**Table 1:** Description of the 11 patients included in the study, detailing all of the variants previously detected

Patient	Phenotype	Other features	Gene	Identification	Genetic variant	Protein variant	Location	*In silico* prediction	cvg	Status
1	TCA (male)	Familial, non syndromic	*RET*	CS065611	c.1263 + 2 T > A	-	10:g.43604680 T > A; intronic	Splicing site	268X	Detected
			*NTRK3*	rs139392904	c.1933C > T	p.Arg645Cys	15:g.88472622G > A; CDS	−/+	311X	Detected
			*EDN3*	rs11570344	c.559_560insA	p.Glu187Glu	20:g.57897443_57897444insA; CDS	Inframe insertion	404X	Detected
2	L-HSCR (male)	Sporadic, non syndromic	*SEMA3D*	rs370785183	c.1901G > A	p.Arg634Gln	7:g.84636125C > T; CDS	−/+	90X	Detected
3	L-HSCR (male)	Sporadic, non syndromic	*PROK1*	rs62623571	c.142C > T	p.Arg48Trp	1:g.110996652C > T; CDS	−/+	152X	Detected
4	S-HSCR (male)	Sporadic, non syndromic	*RET*	rs17158558	c.2944C > T	p.Arg982Cys	10:g.43620335C > T; CDS	−/+	146X	Detected
			*SOX10*	No	c.153delC	p.Gly52Alafs56Ter	22:g.38379639delG; CDS	STOP gained	46X	Detected
5	S-HSCR (male)	Sporadic, non syndromic	*PROKR1*	rs373101730	c.1063A > T	p.Lys354Asn	2:g.68882589A > T; CDS	−/+	2332X	Detected
6	S-HSCR (male)	Sporadic, non syndromic	*PSPN*	rs199881560	c.271C > T	p.Arg91Cys	19:g.6375505G > A; CDS	+/+	13X	Detected
			*RET*	CX065873	c.1776G > A	p.Gly592Gly_Gly593Ter	10:g.43609020GG > AT; CDS	STOP gained	119X	Detected
7	TCA (male)	Sporadic, non syndromic	*NTRK3*	No	c.1229 + 21delTCC	-	15:g.88670477-79delGGA; intronic	Intronic	105X	Detected
			*PROKR2*	rs78861628	c.802C > T	p.Arg268Cys	20:g.5283039G > A; CDS	+/+	5266X	Detected
8	L-HSCR (male)	Sporadic, non syndromic	*NTF3*	rs1805149	c.226G > A	p.Gly76Arg	12:g.5603607G > A; CDS	−/−	1247X	Detected
9	NA (male)	Sporadic, non syndromic	*NRTN*	No	c.199G > A	p.Ala67Thr	19:g.5827789G > A; CDS	−/+	7X	Detected
			*NRTN*	CM981393	c.258 G > T	p.Ala96Ser	19:g.5827876G > T; CDS	−/−	0X	Not detected
10	L-HSCR (female)	Sporadic, non syndromic	*PHOX2B*	No	c.393_410del18bp^(1)^	p.Ala131_Leu136del	4:g.41749386_41749404delGGTCGATCTTCAGGGCCA; CDS	Framehisft mutation	163X	Detected
			*GDNF*	rs36119840	c.277C > T	p.Arg93Trp	5:g.37816112G > A; CDS	+/+	704X	Detected
11	S-HSCR (male)	Sporadic, syndromic (X-linked hydrocephalus)	*L1CAM*	CM981156	c.2077G > A	p.Gly693Arg	X:g.153132856C > T; CDS	+/+	221X	Detected

*TCA* total colonic aganglionosis, *L-HSCR* long-segment HSCR, *S-HSCR* short-segment HSCR, *NA* not available data, *Chr* chromosome, *cvg* mean coverage, *CDS* coding DNA sequence

(1) c.93_410delGGTCGATCTTCAGGGCCA

*In silico* prediction: aminoacidic changes (−/−: benign for SIFT and Polyphen; −/+: damaging in one; +/+: damaging in both)

We also included a group of 200 healthy control subjects comprising unselected, unrelated, race, age, and sex-matched individuals, to determine the allelic frequency of the new variants in our population.

All subjects underwent peripheral blood extraction for genomic DNA isolation using MagNA Pure LC system (Roche, Indianapolis, IN) according to the manufacturer’s instructions. DNA samples were stored at −80 °C until needed for further analyses.

### Ethics statement

A written informed consent was obtained from all the participants for clinical and molecular genetic studies. The study was approved by the Ethics Committee for clinical research in the University Hospital Virgen del Rocío (Seville, Spain) and complies with the tenets of the declaration of Helsinki.

### Design of the capture panel and estimation of panel yield

A capture panel of HSCR related genes was designed by our group and the final file was submitted to Roche NimbleGen (Roche NimbleGen Inc., Madison, WI, USA) to synthesize the hybridization probes. The probes covered 235 regions (exons and closer introns) of 26 known HSCR genes with a total of 44.196 bp in design region (Additional file 1). Flanking sequences were also detected by our sequencer, raising the number to 62.515 bp.

Sensitivity and specificity of the panel were calculated according to methods previously described [21]. Regarding sensitivity, it was calculated as the percentage of variants previously detected by conventional Sanger sequencing that the panel is able to detect. This was tested with 18 variants previously diagnosed (SNVs, insertions and deletions). The specificity was calculated as the percentage of variants detected by the panel that conform to sequencing quality controls and are validated by Sanger sequencing, and therefore are true variants.

### DNA library preparation and targeted sequencing

Library preparation was performed according to the manufacturer’s protocol [SeqCap_EZ_Library_LR_Guide_v2.0 and SeqCap_EZ_LR_DoubleCapture_Rapid_v1p4_2 protocols (Roche NimbleGen Inc., Madison, WI, USA)]. Briefly, 500 ng of genomic DNA was fragmented among 500–1500 bp, then end repaired and ligated with adaptors. The library was amplified by precapture linker-mediated PCR (LM-PCR). After purification, 1 μg LM-PCR product was hybridized to custom designed SeqCap EZ Library (Roche NimbleGen, Madison, WI, USA). After washing, amplification was performed with post-capture LM-PCR. This process was repeated twice. The final concentration of each captured library was measured with Quant-iT PicoGreen dsDNA Assay Kit (Invitrogen, Carlsbad, CA, USA) and diluted at 10^6^ molecules/μl. To perform the emulsion PCR, a 0.7 molecule per bead ratio was chosen. After enrichment, a maximum of 250.000 beads were sequenced on 454-GS Junior (Roche) sequencer according to the manufacturer’s protocol (Sequencing Method Manual GS Junior, Titanium Series).

### Bioinformatic analyses of the sequencing results

Sequencing reads were aligned to human hg19 reference by GS Reference Mapper software (Roche, version 2.7). Improperly mapped reads were filtered out with the SAMtools package. The BEDtools package was applied to analyze the coverage and the percentage of covered bases. Variant calling was performed with GATK (Genome Analysis Toolkit, version 1.4 for SNVs and 1.0 for INDELs). A minimum of 6X coverage was required for every detected variant; at least 25 % of total reads were needed to support the variant allele and variants with a disequilibrium between forward or reverse < 15 % were removed. Sequence variation annotation was performed using VARIant ANalysis Tool (version 2.1.0) [22]. Annotated variants present in NCBI dbSNP [23] and 1000 Genomes project [24] databases with a minor allele frequency (MAF) > 0.05 were discarded. The remaining variants were compared with human mutation databases such as HGMD [25] and ClinVar [26], to detect known disease-associated variants previously identified by Sanger method. Additional novel sequence variants identified were further prioritized considering their inheritance and type of changes. Candidate variants were obtained based on two criteria:

1. New variants only present in one patient:
  In a first step of the analysis, we discarded variants registered on Ensembl [27] and dbSNP databases. Only exonic and closer intronic regions were selected. All new detected variants were searched in 1000 Genomes and Exome Variant Server [28] to confirm their status of “new variant”.
2. Variants registered in databases:
  Variants with MAF < 0.05 present in Biomart [29] and Variant Effect Predictor [27] were considered. All data were managed with the online web tool Galaxy Project [30–32].

### Assessment of the pathogenicity of variants

The *in silico* prediction tools used were: SIFT [33] and PolyPhen2 [34], to establish the pathogenicity of amino acidic changes; the ENCODE Project [35] to determine the location of variants in regulatory regions; The Berlekey Drosophila Genome project [36], to study splice-site changes; MUpro [37] and I-Mutant2.0 [38] for prediction of protein stability and UniProt [39] to determine the protein domains where the variants were located.

### Criteria to select patients after NGS analyses for further discussion

After NGS analyses, we selected patients based on their new variants detected by this study, in compilation with their previous known genetic background. We excluded those ones who: 1) carry one or several previously described variant(s) that could explain the phenotype of the patient and/or 2) the new variants detected in this study were predicted as benign or they were located at regulatory regions which would require additional studies to ascertain their role in the gene function.

### Sanger validation and segregation analyses

All putative HSCR-related variants and 4 panel regions with insufficient coverage by NGS were validated by Sanger sequencing. DNA sequences were obtained from Ensembl and Primer3 [40, 41] was used for primer design (data and conditions available under request). The products were sequenced by an automated sequencer 3730 DNA analyzer (Applied Biosystems®). Variants were analyzed with the program DNASTAR® Lasergene 8 SeqMan Pro™ (DNAstar, Madison, WI) [42]. All variants were tested for segregation in all available family members by Sanger sequencing and analyzed in a group of 200 healthy control subjects.

Dataset was submitted to the European Nucleotide Archive with an accession number PRJEB7384.

## Results and discussion

### Panel yield

The average percentage of covered bases was 97 % and the median percentage of reads on target of our panel was 82.5 %. The high mean coverage obtained (422X) could be explained by the small size of the panel (less than 50.000 base pairs) (Table 2). From the 235 regions contained in the panel, 231 regions had a minimum coverage above 6X. Moreover, 91.3 % of bases had coverage above 20X. Both sensitivity and specificity were of 94 and 82.8 % respectively.

**Table 2:** Summary of statistics of targeted sequencing in our patients

Patient	N° reads	Reads on target (%)	Covered bases (%)	cvg
1	203871	84.4	97.3	445X
2	165770	84.9	96.7	358X
3	169514	83.4	97.6	343X
4	131085	80.8	96.7	252X
5	241836	83.7	96.8	492X
6	236746	83.4	98.0	611X
7	239404	84.0	97.3	553X
8	227925	84.1	97.3	522X
9	165282	71.5	97.5	279X
10	178037	83.5	96.9	346X
11	215702	83.9	96.9	446X
Mean	197742.9	82.5	97.2	422X

*cvg* mean coverage

### Validation of the panel and detection of new variants

The two main goals of this approach were both the validation of our panel, using variants previously identified by Sanger method in our series of patients (Table 1), and the discovery of new variants that could help to further define the complex genetic basis of the pathology in each patient (Table 3). An average of 200 different SNVs was detected in each patient. After the application of stringent filter criteria, a range of 1 to 4 candidate variants per patient were selected. The SNV validation rate was 88 %. In addition, 6 INDELs were selected for further analysis and 3 were validated by Sanger.

**Table 3:** New variants detected by NGS-based targeted sequencing in all patients

Patient	Gene	Chr position	ID	Changes	MAF	*In silico* prediction
1	*ECE1*	1:g.21573855 A > G	rs1076669	c.1013C > T/p.Thr338Ile	0.04	- / -
	*ECE1*	1:g.21551614 G > A	rs3026905	c.2004 + 129C > T	0.02	-
2	*GFRA3*	5:g.137588322 C > T	-	c.*335 G > A	<0.01	Low activity region
	*RET*	10:g.43600210delGCACAGTCA	rs546164092	c.625 + 2134delGCACAGTCA	0.004	Enhancer
	*RET*	10:g.43600325delCC	rs144431581	c.625 + 2244delCC	>0.05	Enhancer
	*GFRA1*	10:g.117884822 T > A	-	c.665A > T/p.Gln222Leu	<0.01	- / +
3	*SEMA3D*	7:g.84651849 G > T	rs141893504	c.1272C > A/p.His424Gln	<0.01	+ / +
	*GFRA1*	10:g.118030415 A > T	rs8192662	c.253 T > A/p.Tyr85Asn	0.02	+ / +
	*EDNRB*	13:g.78493201 C > T	-	c.-51-442 C > T	<0.01	Promoter
	*GDNF*	5:g.37835932 G > A	-	c.-26-1008 G > A	-	-
4	*ECE1*	1:g.21564631 C > T	rs141146885	c.1376G > A/p.Ser459Asn	<0.01	- / -
5	*PHOX2B*	4:g.41749629 A > G	rs191239994	c.242-76 T > C	<0.01	Enhancer
	*SEMA3C*	7:g.80378343 T > A	rs201228749	c.1713A > T/p.Ala571Ala	-	New splicing site
	*GFRA2*	8:g.21640172 C > T	-	c.280C > T/p.Arg94Cys	<0.01	+ / +
6	*PHOX2B*	4:g.41747630 G > A	rs186778106	c.*194C > A	0.01	Enhancer
	*NRG1*	8:g.32406656 C > T	rs148585725	c.100 + 312C > T	0.01	Promoter /CTCF binding site
	*NRG1*	8:g.32617713C > T	rs79223941	c.1086-5C > T	0.01	-
7	*NRG1*	8:g.32611970 G > T	rs74942016	c.772G > T/p.Val258Leu	0.02	+ / +
8	*GFRA1*	10:g.118030415 A > T	rs8192662	c.253 T > A/p.Tyr85Asn	0.02	+ / +
10	*NRG1*	8:g.32611970 G > T	rs74942016	c.772G > T/p.Val258Leu	0.02	+ / +
	*ECE1*	1:g.21571475 G > A	rs28368004	c.1242 + 7C > T	0.01	CTCF binding site
	*GFRA1*	10:g.118031734 G > C	rs45568534	c.-193C > G	0.01	Enhancer
11	*EDNRB*	13:g.78492763 G > C	-	c.*988C > G	-	Predicted promoter
	*NTF3*	12:g.5541624 C > G	rs71578945	c.18 + 116C > G	<0.01	Enhancer

Description of all new variants found in this study, except for patient 9, who was not found to carry any new additional variant by NGS

*In silico* prediction: amino acidic changes (− / −) benign for SIFT and Polyphen; (− / +) damaging in one; (+ / +) damaging in both

Non-coding regions evaluated by the ENCODE project database

After exclusion of all false-positives, validation and segregation analyses were performed. A total of 13 new different coding variants potentially involved in HSCR were obtained and only 5 were previously described. In addition, we identified 11 new non-coding variants in regulatory regions, most of them with an *in silico* prediction of affecting enhancer, promoter and/or CCCTC-binding sites (CTCF) (Table 3). It has been previously determined the critical role of regulatory variants in intronic regions, mainly a common *RET* variant (rs2435357; 10:g.43086608 T > C) located in a gut-specific *RET* enhancer element in intron 1 [8]. A higher focus on these kind of variants would be interesting in further studies because most of NGS targeted studies are limited to present coding variants, but non-coding variants located in regulatory regions can also affect the gene expression and thereby, the phenotype of disease.

### Contributions of new variants

**Fig. 1:**
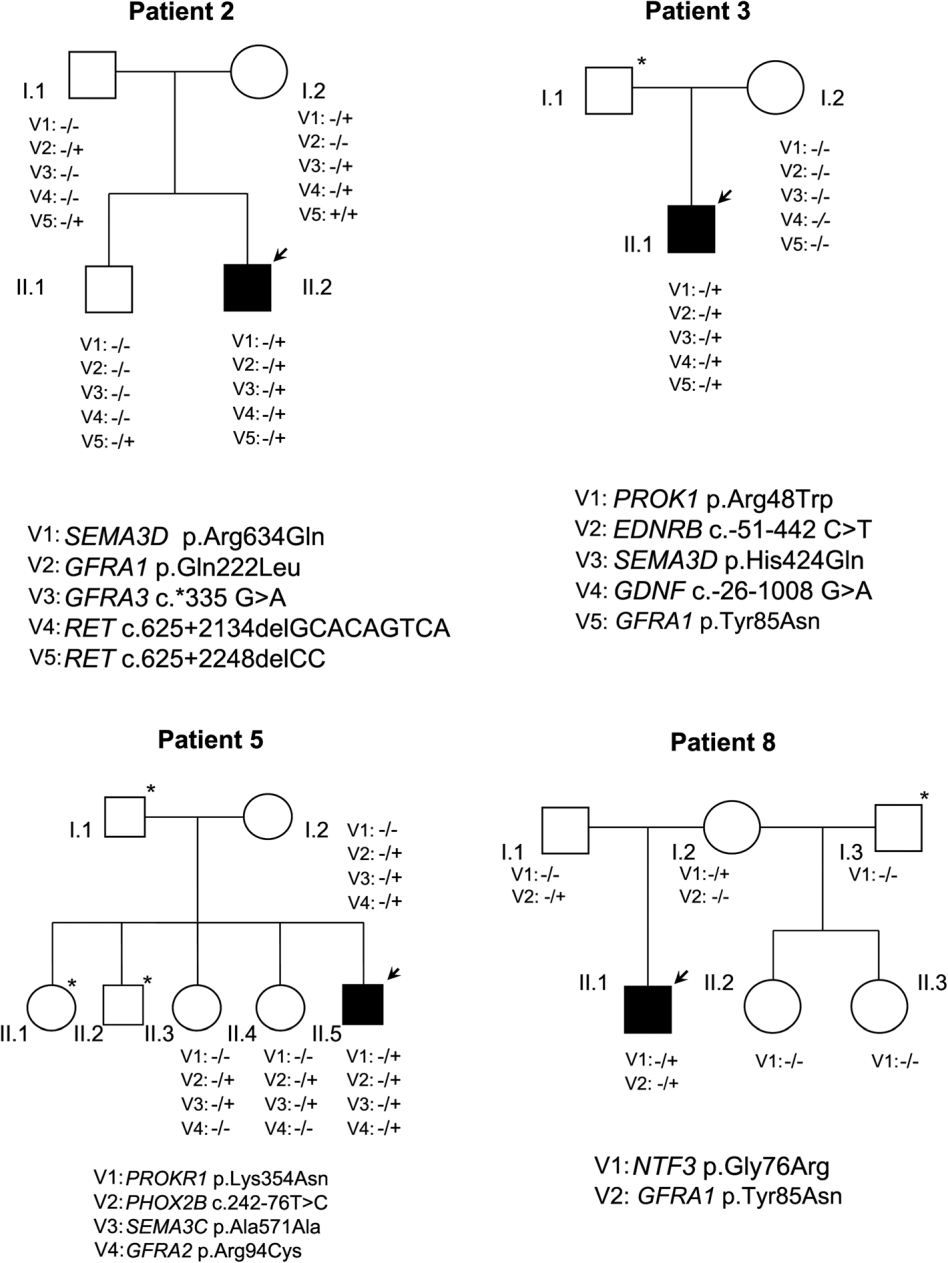
Family trees of patients 2, 3, 5 and 8. All previously identified variants and the new ones found in this study were included. Symbols: V = variant; arrow = patient included in the study; genotypes: − = wildtype allele; + = non-standard allele; * = not available DNA

The previously known genetic background of our patients, together with the new variants found, allowed us to define more precisely the molecular basis of the disease in 4 of the 11 patients (numbers 2, 3, 5 and 8) (Fig. 1). The remaining 7 cases were not found to carry any new relevant variant that contributed to better explain their phenotype.

Of note, patients 2, 3 and 5 presented alterations in class-III semaphorin and in GFRα receptor genes simultaneously (Fig. 1 and Table 3). Several families of molecules implicated in attractive and repulsive guidance are involved in axon guidance, such as semaphorins and GDNF. Some crucial mechanisms in HSCR are mediated by GDNF, which requires GFRα1 as a co-receptor for optimal ligand binding and activation, and both act as chemoattractants to promote neurite outgrowth [43, 44]. Based on these previous studies, we hypothesize that an additive effect of variants in both semaphorins (involved in cell migration) and GFRα receptors (related to proliferation and cell survival) may act as modifier in HSCR. Recently, it has been demonstrated that Sema3C/3D signaling is an evolutionarily conserved regulator of ENS development and its dys-regulation leads to enteric aganglionosis [45]. Paratcha and Charoy functionally showed the interplay between GDNF and GFRα, as well as SEMAs and GDNF signaling during axon guidance, respectively. Charoy et al. analyzed single and double mutant mouse models to confirm that gdnf is the principal trigger of Sema3B, acting with NrCAM. In addition, genetic and *in vitro* experiments provide evidence that this gdnf effect is mediated by NCAM/GFRα1 signaling. In conclusion, our observations suggest a potential combination of variants in these genes that could contribute to disease, based on the demonstrated interplay among this type of molecules, although further functional and statistical studies would be required for confirmation.

Patient 2 (L-HSCR) presented a previously known *SEMA3D* p.Arg634Gln variant, with maternal inheritance and a damaging/benign *in silico* prediction by SIFT/Polyphen respectively. We have identified four new heterozygous variants. The most relevant one was *GFRA1* p*.*Gln222Leu, with paternal inheritance and an *in silico* prediction of tolerated/possibly damaging (Fig. 1, Tables 3 and 4). This patient could fit in the additive model proposed for HSCR based on the paternal and maternal inheritance of his variants. As we mentioned before, the joint effect of variations in *SEMAs* and *GFRAs* genes could help to gain insight into the genetic basis of the disease in this patient.

**Table 4:** *In silico* predictions of functional effect for most relevant variants in patients 2, 3, 5 and 8

Patient	Variants	SIFT/Polyphen	MUpro	I-Mutant 2.0	Uniprot
2	*SEMA3D* p.Arg634Gln	Damaging (S = 0.05)/Benign (S = 0.02)	Decreased stability (CS = −0.53)	Decreased stability (RI = 8)	Domain Ig-Like C2 type
	*GFRA1* p*.*Gln222Leu	Tolerated (S = 0.07)/Possibly damaging (S = 0.46)	Increased stability (CS = 0.99)	Increased stability (RI = 7)	-
3	*PROK1* p.Arg48Trp	Damaging (S = 0)/Probably damaging (S = 1.0)	Decreased stability (CS = −0.99)	Decreased stability (RI = 6)	-
	*SEMA3D* p.His424Gln	Damaging (S = 0)/Probably damaging (S = 1.0)	Decreased stability (CS = −0.79)	Decreased stability (RI = 8)	SEMA
	*GFRA1* p.Tyr85Asn	Damaging (S = 0.05)/Possibly damaging (S = 0.88)	Decreased stability (CS = −1)	Decreased stability (RI = 7)	-
5	*PROKR1* p.Lys354Asn	Damaging (S = 0.08)/Probably damaging (S = 0.99)	Decreased stability (CS = −0.78)	Increased stability (RI = 4)	-
	*GFRA2* p. Arg94Cys	Damaging (S = 0.01)/Probably damaging (S = 1.0)	Decreased stability (CS = −0.98)	Decreased stability (RI = 5)	-
8	*NTF3* p.Gly76Arg	Benign (S = 1.0)/Benign (S = 0)	Decreased stability (CS = −0.84)	Decreased stability (RI = 4)	Propeptide
	*GFRA1* p.Tyr85Asn	Damaging (S = 0.05)/Possibly damaging (S = 0.88)	Decreased stability (CS = −1)	Decreased stability (RI = 7)	-

*S* score, *CS* confidence score, *RI* reliability index

(−) = not located at a specific domain

Patient 3 (L-HSCR) was previously known to carry *PROK1* p.Arg48Trp variant with a probably damaging *in silico* prediction. Our group previously published that *PROK1* may participate in a complementary signalling to the RET/GFRα1/GDNF pathway, giving support to the proliferation/survival and differentiation of precursor cells during ENS development [14]. From the new variants detected in this patient (Fig. 1, Tables 3 and 4) and based on both the *in silico* probably damaging prediction and the described interconnection among these genes, we suggest that *GFRA1* p.Tyr85Asn could interact with *SEMA3D* p.His424Gln and thus*,* together with *PROK1* p.Arg48Trp variant, would contribute to better understand the genetic basis of HSCR in this case.

Patient 5 (S-HSCR) had a previously known variant in *PROKR1* p.Lys354Asn with an *in silico* prediction of tolerated/probably damaging. In this study, he was found to carry a synonymous variant in *SEMA3C* p.Ala571Ala, predicted as pathogenic due to the alteration of an exonic splicing enhancer site (ESE) (Fig. 1, Tables 3 and 4). The ESE sites are targeted essentially by Serine/Arginine-rich proteins defining the splice-sites within the exons [46]. Genomic variations causing aberrant splicing may represent up to 50 % of all mutations that lead to gene dysfunction and pathology [47–49]. Furthermore, patient 5 showed a new variant in *GFRA2* p. Arg94Cys with damaging prediction (Table 4). *GFRA2* had been previously evaluated as a candidate gene for HSCR in just one previous study [50]. Six coding variants were identified, but only 2 led to an amino acidic change at protein level. Both changes were located at the C-terminus of *GFRA2*, a region which is not crucial for GFRα binding to either RET or GDNF family members. The authors concluded that *GFRΑ2* variants were unlikely to represent a common genetic cause or modifier of the HSCR phenotype. In contrast, our analyses have revealed a heterozygous C > T variant in exon 2 of *GFRA2* gene, that causes a highly conserved arginine-94 residue substitution to a cysteine residue in the cysteine rich domain of the receptor. *In silico* predictions suggest that p.Arg94Cys variant would decrease the stability of protein structure and it could be a non-neutral change. Our results would suggest that *GFRA2* might be reconsidered as a candidate gene for HSCR.

Finally, patient 8 (L-HSCR), who presented a known variant in *NTF3* p.Gly76Arg (benign prediction), was found to carry *GFRA1* p.Tyr85Asn (pathogenic prediction) (Fig. 1, Tables 3 and 4) as well. Different studies have described the association of polymorphisms with HSCR, which might suggest the possibility to consider *GFRA1* p.Tyr85Asn as a putative susceptibility factor in this patient [51, 52]. However, to confirm this hypothesis, further case-control studies in additional series of patients are required.

## Conclusions

We have validated the high capacity of the NGS targeted sequencing to detect SNVs, which accounts for most of the variants, pathogenic or not, in the genes included in the panel. Many of the possible insertions and deletions detected by NGS with the 454 GS-Junior (around a thousand for each patient) were false positives due to the limitations of the technique to detect this type of variants [53]. Our study also provides a higher coverage of the included regions and a manageable amount of data to be analyzed than other studies [54]. Additional newly discovered HSCR-linked genes could be included in panels similar to ours due to their flexibility. Also, this design could be adapted to different sequencing platforms.

Our validated NGS panel has resulted in a fast, effective and easy method to characterize the genetic background in our patients and to identify new variants that could be associated to HSCR. Our results expand the previously known set of variants carried by these patients and further support the feasibility of using NGS targeted sequencing in diseases with complex genetic basis such as HSCR. Moreover, this technique may help in the understanding of the genetic and molecular basis of the disease, providing a new tool in clinical practice to simultaneously analyze many genes as well as to identify several molecular events contributing to the phenotype.

## Additional file

Additional file 1:
**Detailed description of covered regions in our panel of genes.** (XLS 53 kb)

## References

[1] Amiel J, Sproat-Emison E, Garcia-Barcelo M, Lantieri F, Burzynski G, Borrego S (2008). Hirschsprung disease, associated syndromes and genetics: a review. J Med Genet.

[2] Barlow A, de Graaff E, Pachnis V (2003). Enteric nervous system progenitors are coordinately controlled by the G protein-coupled receptor EDNRB and the receptor tyrosine kinase RET. Neuron.

[3] Borrego S, Ruiz-Ferrer M, Fernandez RM, Antinolo G (2013). Hirschsprung’s disease as a model of complex genetic etiology. Histol Histopathol.

[4] Chakravarti ALS (2001). Hirschsprung disease.

[5] Druckenbrod NR, Epstein ML (2009). Age-dependent changes in the gut environment restrict the invasion of the hindgut by enteric neural progenitors. Development.

[6] Borrego S, Wright FA, Fernandez RM, Williams N, Lopez-Alonso M, Davuluri R (2003). A founding locus within the RET proto-oncogene may account for a large proportion of apparently sporadic Hirschsprung disease and a subset of cases of sporadic medullary thyroid carcinoma. Am J Hum Genet.

[7] Emison ES, Garcia-Barcelo M, Grice EA, Lantieri F, Amiel J, Burzynski G (2010). Differential contributions of rare and common, coding and noncoding Ret mutations to multifactorial Hirschsprung disease liability. Am J Hum Genet.

[8] Emison ES, McCallion AS, Kashuk CS, Bush RT, Grice E, Lin S (2005). A common sex-dependent mutation in a RET enhancer underlies Hirschsprung disease risk. Nature.

[9] Borrego S, Fernandez RM, Dziema H, Niess A, Lopez-Alonso M, Antinolo G (2003). Investigation of germline GFRA4 mutations and evaluation of the involvement of GFRA1, GFRA2, GFRA3, and GFRA4 sequence variants in Hirschsprung disease. J Med Genet.

[10] Fernandez RM, Sanchez-Mejias A, Mena MD, Ruiz-Ferrer M, Lopez-Alonso M, Antinolo G (2009). A novel point variant in NTRK3, R645C, suggests a role of this gene in the pathogenesis of Hirschsprung disease. Ann Hum Genet.

[11] Jiang Q, Turner T, Sosa MX, Rakha A, Arnold S, Chakravarti A (2012). Rapid and efficient human mutation detection using a bench-top next-generation DNA sequencer. Hum Mutat.

[12] Ruiz-Ferrer M, Fernandez RM, Antinolo G, Lopez-Alonso M, Borrego S (2008). NTF-3, a gene involved in the enteric nervous system development, as a candidate gene for Hirschsprung disease. J Pediatr Surg.

[13] Ruiz-Ferrer M, Torroglosa A, Luzon-Toro B, Fernandez RM, Antinolo G, Mulligan LM (2011). Novel mutations at RET ligand genes preventing receptor activation are associated to Hirschsprung’s disease. J Mol Med (Berl).

[14] Ruiz-Ferrer M, Torroglosa A, Nunez-Torres R, de Agustin JC, Antinolo G, Borrego S (2011). Expression of PROKR1 and PROKR2 in human enteric neural precursor cells and identification of sequence variants suggest a role in HSCR. PLoS One.

[15] Tang CS, Ngan ES, Tang WK, So MT, Cheng G, Miao XP (2012). Mutations in the NRG1 gene are associated with Hirschsprung disease. Hum Genet.

[16] DNA Sequencing Costs: Data from the NHGRI Genome Sequencing Program (GSP). [http://www.genome.gov/sequencingcosts/]

[17] Schuster SC (2008). Next-generation sequencing transforms today’s biology. Nat Methods.

[18] Metzker ML (2010). Sequencing technologies - the next generation. Nat Rev Genet.

[19] Mardis ER (2008). Next-generation DNA sequencing methods. Annu Rev Genomics Hum Genet.

[20] Gui H, Bao JY, Tang CS, So MT, Ngo DN, Tran AQ (2014). Targeted next-generation sequencing on hirschsprung disease: a pilot study exploits DNA pooling. Ann Hum Genet.

[21] De Schrijver JM, De Leeneer K, Lefever S, Sabbe N, Pattyn F, Van Nieuwerburgh F (2010). Analysing 454 amplicon resequencing experiments using the modular and database oriented variant identification pipeline. BMC Bioinformatics.

[22] Medina I, De Maria A, Bleda M, Salavert F, Alonso R, Gonzalez CY (2012). VARIANT: command line, web service and Web interface for fast and accurate functional characterization of variants found by next-generation sequencing. Nucleic Acids Res.

[23] dbSNP Short Genetics Variations. http://www.ncbi.nlm.nih.gov/SNP/.

[24] Abecasis GR, Auton A, Brooks LD, DePristo MA, Durbin RM, Genomes Project C (2012). An integrated map of genetic variation from 1,092 human genomes. Nature.

[25] Stenson PD, Ball EV, Mort M, Phillips AD, Shiel JA, Thomas NS (2003). Human Gene Mutation Database (HGMD): 2003 update. Hum Mutat.

[26] Landrum MJ, Lee JM, Riley GR, Jang W, Rubinstein WS, Church DM (2014). ClinVar: public archive of relationships among sequence variation and human phenotype. Nucleic Acids Res.

[27] Cunningham F, Amode MR, Barrell D, Beal K, Billis K, Brent S (2015). Ensembl 2015. Nucleic Acids Res.

[28] Exome Variant Server. Available: http://evs.gs.washington.edu/EVS/.

[29] Kasprzyk A (2011). BioMart: driving a paradigm change in biological data management. Database (Oxford).

[30] Blankenberg D, Von Kuster G, Coraor N, Ananda G, Lazarus R, Mangan M (2010). Galaxy: a web-based genome analysis tool for experimentalists. Curr Protoc Mol Biol.

[31] Giardine B, Riemer C, Hardison RC, Burhans R, Elnitski L, Shah P (2005). Galaxy: a platform for interactive large-scale genome analysis. Genome Res.

[32] Goecks J, Nekrutenko A, Taylor J, Galaxy T (2010). Galaxy: a comprehensive approach for supporting accessible, reproducible, and transparent computational research in the life sciences. Genome Biol.

[33] Ng PC, Henikoff S (2001). Predicting deleterious amino acid substitutions. Genome Res.

[34] Adzhubei IA, Schmidt S, Peshkin L, Ramensky VE, Gerasimova A, Bork P (2010). A method and server for predicting damaging missense mutations. Nat Methods.

[35] Consortium EP (2012). An integrated encyclopedia of DNA elements in the human genome. Nature.

[36] Reese MG, Eeckman FH, Kulp D, Haussler D (1997). Improved splice site detection in Genie. J Comput Biol.

[37] Cheng J, Randall A, Baldi P (2006). Prediction of protein stability changes for single-site mutations using support vector machines. Proteins.

[38] Capriotti E, Fariselli P, Casadio R (2005). I-Mutant2.0: predicting stability changes upon mutation from the protein sequence or structure. Nucleic Acids Res.

[39] UniProt C (2014). Activities at the Universal Protein Resource (UniProt). Nucleic Acids Res.

[40] Koressaar T, Remm M (2007). Enhancements and modifications of primer design program Primer3. Bioinformatics.

[41] Untergasser A, Cutcutache I, Koressaar T, Ye J, Faircloth BC, Remm M (2012). Primer3--new capabilities and interfaces. Nucleic Acids Res.

[42] Lasergene8-Seqman Pro. Available: http://www.dnastar.com/t-seqmanpro.aspx.

[43] Charoy C, Nawabi H, Reynaud F, Derrington E, Bozon M, Wright K (2012). gdnf activates midline repulsion by Semaphorin3B via NCAM during commissural axon guidance. Neuron.

[44] Paratcha G, Ledda F (2008). GDNF and GFRalpha: a versatile molecular complex for developing neurons. Trends Neurosci.

[45] Jiang Q, Arnold S, Heanue T, Kilambi KP, Doan B, Kapoor A (2015). Functional loss of semaphorin 3C and/or semaphorin 3D and their epistatic interaction with ret are critical to Hirschsprung disease liability. Am J Hum Genet.

[46] Blencowe BJ (2000). Exonic splicing enhancers: mechanism of action, diversity and role in human genetic diseases. Trends Biochem Sci.

[47] Cartegni L, Chew SL, Krainer AR (2002). Listening to silence and understanding nonsense: exonic mutations that affect splicing. Nat Rev Genet.

[48] Piton A, Jouan L, Rochefort D, Dobrzeniecka S, Lachapelle K, Dion PA (2013). Analysis of the effects of rare variants on splicing identifies alterations in GABAA receptor genes in autism spectrum disorder individuals. Eur J Hum Genet.

[49] Ramser J, Abidi FE, Burckle CA, Lenski C, Toriello H, Wen G (2005). A unique exonic splice enhancer mutation in a family with X-linked mental retardation and epilepsy points to a novel role of the renin receptor. Hum Mol Genet.

[50] Vanhorne JB, Gimm O, Myers SM, Kaushik A, von Deimling A, Eng C (2001). Cloning and characterization of the human GFRA2 locus and investigation of the gene in Hirschsprung disease. Hum Genet.

[51] Liang CM, Ji DM, Yuan X, Ren LL, Shen J, Zhang HY (2014). RET and PHOX2B genetic polymorphisms and Hirschsprung’s disease susceptibility: a meta-analysis. PLoS One.

[52] Wang Y, Wang J, Pan W, Zhou Y, Xiao Y, Zhou K (2013). Common genetic variations in Patched1 (PTCH1) gene and risk of hirschsprung disease in the Han Chinese population. PLoS One.

[53] Zhang J, Chiodini R, Badr A, Zhang G (2011). The impact of next-generation sequencing on genomics. J Genet Genomics.

[54] Christodoulou K, Wiskin AE, Gibson J, Tapper W, Willis C, Afzal NA (2013). Next generation exome sequencing of paediatric inflammatory bowel disease patients identifies rare and novel variants in candidate genes. Gut.

